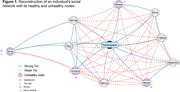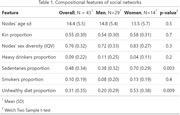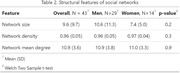# Exploring Social Networks in the LatAmFINGERS Trial: The Importance of Pre‐Intervention Social Involvement

**DOI:** 10.1002/alz.090874

**Published:** 2025-01-09

**Authors:** Carolina Agata Ardohain Cristalli, Lucia Crivelli, Paulo Caramelli, Francisco Lopera, Ricardo Nitrini, Ana Luisa Sosa, Rosa Maria Salinas‐Contreras, Claudia Kimie Suemoto, Monica Sanches Yassuda, Gustavo Sevlever, Heather M Snyder, Miia Kivipelto, Maria C. Carrillo, Daisy M Acosta, Ana Charamelo, María Isabel Cusicanqui, Nilton Custodio, Carolina Delgado, Lissette Duque, Ivonne Z. Jimenez Velazquez, Jorge M Leon‐Salas, Ricardo Allegri, Ismael Luis Calandri, LatAm‐FINGERS Consortium

**Affiliations:** ^1^ Fleni, Buenos Aires Argentina; ^2^ Fleni, Buenos Aires, CABA Argentina; ^3^ Faculty of Medicine ‐ Universidade Federal de Minas Gerais, Belo Horizonte Brazil; ^4^ Grupo de Neurociencias de Antioquia, Facultad de Medicina, Universidad de Antioquia, Medellín Colombia; ^5^ Universidade de Sao Pablo, Sao Pablo Brazil; ^6^ Dementias Laboratory, National Institute of Neurology and Neurosurgery, Mexico City, DF Mexico; ^7^ University of São Paulo Medical School, São Paulo, São Paulo Brazil; ^8^ University of São Paulo, SAO PAULO, SAO PAULO Brazil; ^9^ Fleni, Buenos Aires, Buenos Aires Argentina; ^10^ Alzheimer’s Association, Chicago, IL USA; ^11^ Theme Inflammation and Aging, Karolinska University Hospital, Stockholm Sweden; ^12^ Universidad Nacional Pedro Henriquez Ureña, Santo Domingo, Distrito Nacional Dominican Republic; ^13^ Departamento de Neuropsicología, Facultad de Medicina‐Hospital de Clínicas, Universidad de la República, Montevideo Uruguay; ^14^ Centro Neurológico Mente Activa, La Paz Bolivia (Plurinational State of); ^15^ Unidad de Investigación, Instituto Peruano de Neurociencias, Lima, Perú, Lima, Lima Peru; ^16^ Universidad de Chile, Santiago, Region Metropolitana Chile; ^17^ Neuromedicenter, Quito Ecuador; ^18^ University of Puerto Rico, School of Medicine, San Juan, Puerto Rico, PR USA; ^19^ Clínica Bíblica Hospital, San José Costa Rica

## Abstract

**Background:**

LatAm‐FINGERS is a non‐pharmacological multicenter randomized clinical trial aimed at preventing cognitive impairment. The intervention advocates for a lifestyle change based on diet, exercise, risk factor control, cognitive training, and socialization. However, the baseline assessment lacks a evaluation of the participants sociability before the intervention. Assessing social activity poses inherent challenges due to its elusive nature. An individual’s social networks encompass the entirety of their personal contacts and the interrelationships among them; it provides an objective way to measure sociability. Our aims are to test the feasibility of a method for assessing sociality based on social network features, to show the baseline state in the trial, and to suggest a standard for gauging intervention effectiveness in this domain.

**Method:**

We designed a pilot study to assess the feasibility of evaluating social networks in 50 participants of LatAm‐FINGERS Argentina. Participants were invited to complete an online Spanish version of the International Social Network Questionnaire. It prompts the participant to identify social contacts (emotional support, companions in activities, and health‐related supporters). Participants are then inquired about the habits of these social contacts and their risk factors. With this information, applying graph theory, we reconstruct the each subject´s network (Figure 1), identify healthy and unhealthy nodes, and estimate structural measures of the network.

**Result:**

The survey demonstrated good applicability, with 43/50 subjects able to independently complete it, while 7 subjects reported technology difficulties. Tables 1 and 2 summarize the structural and compositional characteristics of the subjects' social networks at baseline. A significant number of individuals (53.48%) had social networks where the proportion of family members exceeded 50%. There’s substantial age variability, with a mean deviation of 14.4 years among nodes in each network. 48% of social contacts are sedentary, and 31% have unhealthy diets. Men have a significantly higher proportion of sedentary individuals (p = 0.003) and non‐healthy diets (p = 0.009) in their networks.

**Conclusion:**

Our study demonstrates the feasibility of assessing social networks, revealing significant disparities among individuals and genders. Previous studies highlight the influence of networks on individual risk factors, underscoring the need to incorporate this measure as a mediator of lifestyle changes' effectiveness.